# Editor’s Note

**DOI:** 10.1289/ehp.123-A106

**Published:** 2015-05-01

In the February 2014 issue of *Environmental Health Perspectives*, Albanito et al. retracted their article “G-Protein–Coupled Receptor 30 and Estrogen Receptor-α Are Involved in the Proliferative Effects Induced by Atrazine in Ovarian Cancer Cells” because they discovered that errors were introduced during the sorting of files related to the presentation of the Western blots in Figures 6 and 8 [Environ Health Perspect 122:A42 (2014); doi:10.1289/ehp.11297RET]. There was no evidence that the errors that led to the retraction were intentional, and the authors were forthcoming in acknowledging the errors. Thus, *EHP* agreed to consider a new manuscript on this topic.

The article “Effects of Atrazine on Estrogen Receptor α– and G Protein–Coupled Receptor 30–Mediated Signaling and Proliferation in Cancer Cells and Cancer-Associated Fibroblasts” [Environ Health Perspect 123:493–499; http://dx.doi.org/10.1289/ehp.1408586] contains corrected Western blots as well as new data. This manuscript was handled as a new submission and underwent *EHP*’s standard peer-review process.

